# Description of *Pella maoershanensis* sp. n. (Coleoptera, Staphylinidae, Aleocharinae) associated with *Lasius spathepus* from Guangxi, South China

**DOI:** 10.3897/zookeys.275.4449

**Published:** 2013-03-04

**Authors:** Xiao-Bin Song, Li-Zhen Li

**Affiliations:** 1 Department of Biology, College of Life and Environmental Sciences, Shanghai Normal University, 100 Guilin Road, Xuhui District, Shanghai 200234, P. R. China

**Keywords:** Coleoptera, Staphylinidae, Aleocharinae, *Pella*, South China, myrmecophilous

## Abstract

*Pellamaoershanensis* Song & Li, **sp. n.**, collected from a colony of *Lasius (Dendrolasius) spathepus* in Maoershan Natural Reserve, Guangxi, is diagnosed, described and illustrated. The discovery represents the first record of the genus in South China.

## Introduction

According to the latest catalogue of Lomechusini (Hlaváč et al. 2011), the genus *Pella* Stephens contains 61 species worldwide, among which seven are known from China: *Pella beijingorum* Pace (Beijing), *Pella cooterorum* Maruyama (Beijing, Yunnan), *Pella hlavaci* Maruyama (Beijing), *Pella jureceki* Dvořák (Beijing), *Pella kishimotoi* Maruyama (Hunan), *Pella puetzi* Assing (Yunnan) and *Pella zhoui* Maruyama (Beijing). Members of *Pella* are commonly found in association with the ant genus *Lasius* Fabricius (Maruyama 2006). Recently, the senior author and his colleagues surveyed the staphylinid fauna of the Maoershan Mountain (Guangxi, South China), and collected a large series of an unidentified aleocharine beetle by sifting leaf litter near a nest of *Lasius (Dendrolasius) spathepus*. A closer examination of this material revealed a new species of the genus *Pella*. In this paper we describe the new species, provide illustrations of its major diagnostic features, and briefly discuss the biology.

## Meterials and methods

Specimens were killed with ethyl acetate and preserved in 75% ethanol before dissection;

Photos of habitus were taken with a Canon EOS 50D with an MP-E 65mm Macro Photo Lens.

Head length was measured from the clypeal anterior margin to the occipital constriction; elytral length at the suture from the apex of the scutellum to the elytral posterior margin.

All the types were deposited in the Insect Collection of Shanghai Normal University, Shanghai, China (**SNUC**).

## Taxonomy

### Pella maoershanensis

Song and Li sp. n.

urn:lsid:zoobank.org:act:C88CD3B9-6194-4FF7-8EB3-96EE761E35E4

http://species-id.net/wiki/Pella_maoershanensis

Fig. 1

#### Type material

(24 ♂♂, 28 ♀♀)**.** Holotype: ♂, labeled ‘25°53'03.51"N, 110°29'15.67"E / Maoer shan / (1,150 m). Xingan County / Guilin City / [Guangxi, China] / 24.VII.2012, Song X-B & Hu J-Y // HOLOTYPE [red] / *Pella maoershanensis* sp. n. / Song & Li / det. 2013, SNUC’. Paratypes: 23 ♂♂, 28 ♀♀, same label data as holotype, all bearing the following label: ‘PARATYPE [yellow] / *Pella maoershanensis* sp. n. / Song & Li / det. 2013, SNUC’.

#### Diagnosis.

*Pella maoershanensis* shares with *Pella puetzi* a similar form of male sexual character on the head (Assing 2009). The two species can be readily distinguished by the smaller body size, the distinctly transverse antennomeres VI–X, and different forms of the aedeagal distal crest and ventral process in *Pella maoershanensis*. The new species is also similar to the other species of the *Pella cognata* group in general appearance (Maruyama 2006) but can be readily separated by the sexually modified head in the male.

#### Description.

**Figure 1.**
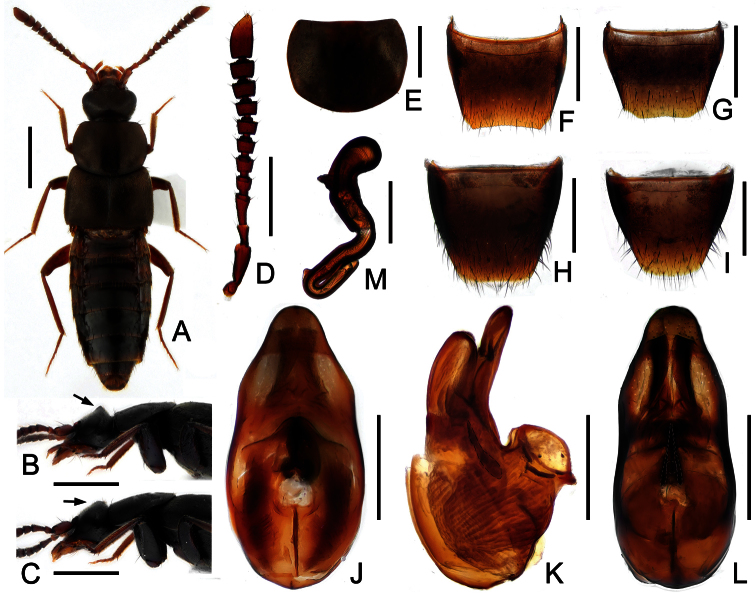
*Pella maoershanensis*. **A** Dorsal habitus **B** Head and pronotum in lateral view, male **C** Head and pronotum in lateral view, female **D** Antenna **E** Pronotum **F** Male tergite VIII **G** Female tergite VIII **H** Male sternite VIII **I** Female sternite VIII **J** aedeagus, in ventral view **K** same, in lateral view **L** same, in dorsal view **M** Spermatheca. Scales (mm): **A** = 2; **B, C** = 1; **D** = 0.25; **E, F, G, H, I** = 0.5; **J, K, L** = 0.3; **M** = 0.2.

Body (Fig. 1A) length: 5.5–5.8 mm. Coloration: fore body brownish; abdomen blackish, with the posterior margins of the segments reddish-brown; leg and antennae reddish-brown.

Head (Fig. 1A) almost 1.2 times as wide as long; widest just behind eyes; surface finely reticulate, covered with short golden setae. Antennae (Fig. 1D) about 2.2 mm long, shorter than head, pronotum and elytra combined; antennomeres VI–X distinctly transverse. Pronotum (Fig. 1E) 1.35 times as wide as long and 1.37 times as wide as head; widest around anterior third, narrowed posteriorly; posterior margin almost rounded; covered with short golden setae, with six macrosetae; hypomera fully visible in lateral view. Elytra (Fig. 1A) about 1.9 times as long as pronotum; covered with short golden setae; humeral angle with one macrosetae. Hind wings fully developed. Abdomen (Fig. 1A) widest at segments IV–V; surface with transverse microsculpture.

Male. Posterior margin of head distinctly angled at middle (Fig. 1B); posterior margin of tergite VIII broadly concave and finely crenulate (Fig. 1F); posterior margin of sternite VIII (Fig. 1H) almost truncate; median lobe of aedeagus as in Figs 1J–L.

Female. Posterior margin of head indistinctly angled at middle (Fig. 1C); tergite VIII (Fig. 1G) and sternite VIII (Fig. 1I) distinctly shorter than that of male; spermatheca as in Fig. 1M.

#### Host ant.

**Figure 2.**
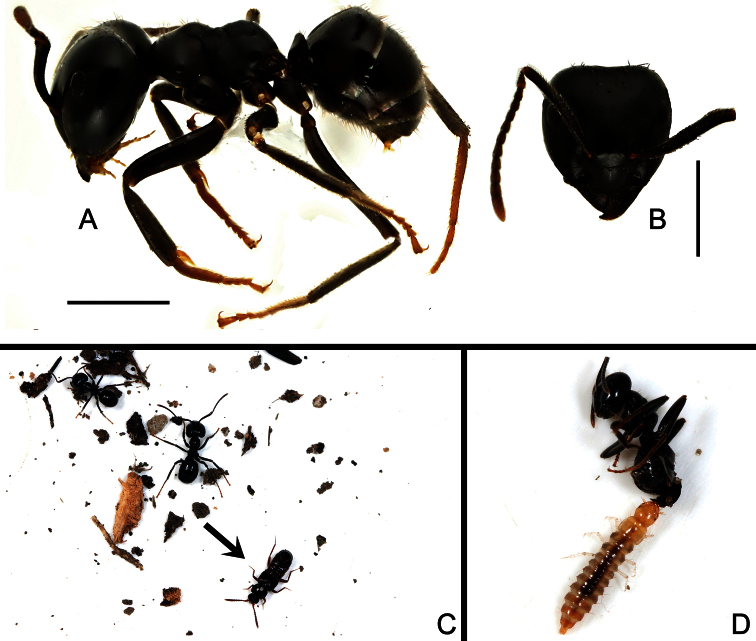
**A** Host ant, habitus in lateral view **B** Same, anterior view of head **C** A living species of *Pella maoershanensis* with host ants**D** A possible larva of *Pella maoershanensis* feeding on a dead worker of host ant. Scales (mm): **A, B** = 1.

*Lasius (Dendrolasius) spathepus* (det. by M. Maruyama) (Figs 2A, B).

#### Biological notes.

Species of *Pella* are usually observed walking around the host’s nest but never appear in it (Maruyama 2006). All individuals of the new species, plus three other aleocharine species were taken by sifting mixed leaf litter around the ant nest (Fig. 2C). Three possible larvae of the new species were taken back to the lab, and were observed to feed on a dead worker of the host ant (Fig. 2D). It’s worth a note that two males and a female of an undescribed *Dendrolasiophilus* Nomura species (Yin pers. comm.) were directly collected from the deep site of the nest.

#### Etymology.

Named after the type locality.

## Supplementary Material

XML Treatment for Pella maoershanensis

## References

[B1] Assing V (2009) New species and additional records of Lomechusini from the Palaearctic region (Coleoptera: Staphylinidae: Aleocharinae). Stuttgarter Beiträge zur Naturkunde Serie A (Biologie), Neue Serie 2: 201-226

[B2] Hlaváč P Newton AF Maruyama M (2011) World catalogue of the species of the tribe Lomechusini (Staphylinidae: Aleocharinae). Zootaxa 3075: 1-151

[B3] Maruyama M (2006) Revision of the Palearctic species of the myrmecophilous genus *Pella* (Coleoptera, Staphylinidae, Aleocharinae). National Science Monographs 32: 1-207

